# Lectures by Dr. Gladstone, F. R. S.

**Published:** 1858-06

**Authors:** 


### Selections.

### LECTURES BY DR. GLADSTONE, F. R. S.,

### ON BONE, ENAMEL, AND THE METALS, ETC., USED IN DENTISTRY.

The following is a summary of the lectures delivered by Dr. Gladstone in the College of Dentists:—

LECTURE 1.

The lecturer commenced by referring to the previous lecture by Mr. Hogg, in which he had shown that, in the words of Prof. Huxley, "the teeth are true dermic structures, formed by the deposition of calcareous matter beneath the basement membrane of a dermic papilla, or that which corresponds with one." Thus far the anatomist and physiologist might proceed; it remained for the chemist to examine the composition of these dental tissues, and the calcareous matter deposited in them.

The tissues themselves were exhibited to the audience by dissolving out the mineral portion of a tooth by hydrochloric acid. This "cartilage" is an organic principle, composed of carbon, hydrogen, nitrogen, and oxygen, with, according to Schlieper, 0.13 per cent. of sulphur. When boiled in water it swells up, and dissolves, yielding, according to the observations of the lecturer, made on human teeth, pure gelatine, and no chondrine. There is also a small quantity of membrane, which is not gelatinized by water, with the blood-vessels, nerve-substance, and a little fat.

The hard portion of the tooth consists of lime, in combination with phosphoric and carbonic acids and fluorine. A little magnesia replaces a portion of the lime. Certain soluble salts are sometimes mentioned in analysis of teeth, but these were considered scarcely to belong to the hard portion, and always occur in very small and varying quantity. On the addition of ammonia to the solution in hydrochloric acid, the original phosphate of lime and flouride of calcium appeared as a flocculent white precipitate; and on the subsequent addition of carbonate of soda, the carbonate of lime was reproduced as a chalky deposit. The following are the average proportions of these constituents, as deduced from the best analysis published:—

| | Bone. | Dentine or Ivory. |
|---|---|---|
| Phosphate of lime | 57 | 65 |
| Carbonate of lime | 8 | 5 |
| Fluoride of calcium | 1 | 1 |
| Phosphate of magnesia | 1 | 1 |
| Cartilage | 33 | 28 |
| | 100 | 100 |

| | Enamel. |
|---|---|
| Phosphate of lime | 85.0 |
| Carbonate of lime | 7.5 |
| Fluoride of calcium | 3.0 |
| Phosphate of magnesia | 1.5 |
| Membrane | 3.0 |
| | 100.0 |

The cement differs from bone merely in containing rather more organic matter. Different individuals, however, differ considerably in the composition of their teeth ; molars, as a rule, contain more mineral substance than incisors ; and as the teeth become older, they change somewhat, principally by the great diminution of the carbonate, as compared with the phosphate of lime. The following table from Lassaigne's experiments was among the diagrams exhibited :

| | Organic Matter. | Phosph. Lime. | Carb. Lime. |
|---|---|---|---|
| Child of one day | 35.0 | 51.0 | 14.0 |
| Child of six years | 28.6 | 60.0 | 11.4 |
| Adult man | 29.0 | 61.0 | 10.0 |
| Man of eighty-one years | 33.0 | 66.0 | 1.0 |

The lecturer then passed on to consider the chemical changes that take place in the teeth. There are changes from within—growth, and repair, besides disease and decay —a subject claiming the most serious attention of the dentist, but one to the knowledge of which chemistry as yet has made no valuable contribution. There are changes from without, dependent on the action of whatever passes into the mouth. The most constant of these agents is the saliva—a secretion from various glands. Now this substance has been shown to differ in composition, as derived from the parotid gland, or from those in and beneath the buccal mucous membrane ; and though analyses, more or less complete, have been published by Wright, Mitscherlich, Berzelius, Tiedemann, Gmelin, Lehmann, Frerichs, Van Setten, Magendie, Bernard, L'Heritier, Rayer, Enderlin, Tilanus, Jacubowitsch, and perhaps others, the lecturer found himself unable to say, with any degree of certainty, the general composition of this liquid. The following was given as perhaps the best average for mixed saliva :—

| | |
|---|---|
| Water | 990.0 |
| Ptyalin | 2.5 |
| Albumen | 1.5 |
| Mucus | 2.0 |
| Soda | 0.8 |
| Phosph. and chlor. of alk. | 2.5 |
| Sulphocyanide of alkali | 0.1 |
| Lime, sulphates, fat, etc. | 0.6 |
| | 1000.0 |

In the normal condition, the saliva is slightly alkaline, as may be tested by reddened litmus paper, or yellow tumeric, which are changed in color by it. This is due, according to some, to carbonate of soda—according to others, to an alkaline phosphate. In any case, such saliva cannot injure the teeth ; but in long fasting, in dyspepsia, inflammatory affections of the stomach, rheumatism, etc., it becomes acid, and though very slightly so, it must, by its constantly bathing the teeth, dissolve more or less of their calcareous substance ; and though this may not generally be the primary cause of caries, it cannot fail to accelerate that action. Food containing much vinegar, or other acids, must also attack the teeth; indeed, a tooth was exhibited which had been partially dissolved by acetic acid. Strongly acid medicines are detrimental in the same way ; and the use of acid dentifrices cannot be too strongly deprecated. The formation of "tartar" was said to be connected with the saliva ; it is very various in composition, consisting mainly of earthy phosphates held together by animal matter. Some calculi taken from the parotid gland were exhibited ; they consisted of carbonate and phosphate of lime, with organic substance.

Dr. Gladstone then proceeded to explain the chemical questions that arise in the treatment of diseases of the teeth. If by decay or fracture the nerve be exposed, it may be burnt, or may be destroyed by powerful chemical agents. Creosote, tannin, tincture of galls, nitric acid, chloride of zinc, salts of cobalt, and other substances, are used for this purpose. Their action was explained and demonstrated by taking the clear white of a fresh egg, which bears a chemical analogy to the albuminous substances that constitute the nerve, and acting upon it by nitric acid, or creosote, whereby it was coagulated just as though it had been boiled. In this way also these chemical agents coagulate, and destroy the integrity of the nerve.

### Lecture 2.

The lecturer commenced by stating, that the deleterious effect of a weak or debilitated stomach, or of an acid character of food, on the teeth, which he had inferred on *a priori* grounds, he had since found to be confirmed by experience, and to be recognised by the profession. He warned dentists against the too abundant use of escharotics, as these powerful agents, while destroying the nerve, have more or less effect on other parts of the tooth. He proceeded to describe the different kinds of stopping which have been employed to fill up cavities in the teeth. It might be naturally supposed, that a substance closely resembling in chemical composition the mineral matter of the tooth, would be best; and accordingly D'Ostermeyer's cement had been devised, consisting of thirteen parts of pure caustic lime, and twelve parts of anhydrous phosphoric acid, mixed together and inserted in the cavity, where they combine to a hard mass. There are, however, great practical difficulties in the use of this; and such earthy stoppings (including the terro-metallic cement, three parts sulphate of lime, and one part peroxide of iron) are apt to fall out. Another idea is acted upon; resins are softened by spirit, and forced into the hole. These serve very well for temporary stoppings, but generally become loose, and of course want entirely the hardness and strength of the original teeth. Gutta percha has lately been found well adapted for this purpose.

A widely different method of stopping, however, is adopted by preference. Metals are employed. The proper solvent for these substances are other metals in a fluid condition, for instance, mercury, or quicksilver. Mercury has also the property of forming alloys with many metals, called amalgams, the physical characters of which were demonstrated by the ammoniacal amalgum. The quicksilver combining with the ammonium swelled to perhaps thirty times its original bulk, and became semi-solid and plastic. In dentistry an amalgam of silver and mercury is much used. The main objection to it arises from the injurious physiological properties of mercury. Softness is a desideratum in metals to enable them to be compressed into every part of the cavity; and this property is possessed to a great extent by some metals, such as potassium. The softest metal, however, which can be actually used, is lead; it is also a cheap metal; but it forms a bad stopping, for it soon becomes covered with a film of oxide, which dissolves in an alkaline liquid, like the saliva, as was shown by experiment, and the taking of small quantities of lead into the system will, in time, produce the fearful disease termed colica pictonum. Lead, also, as was experimentally demonstrated, is blackened by sulphuretted hydrogen, and by those organic principles containing sulphur which form a large portion of our food. Gold is unquestionably the best material for the stopping of teeth under ordinary circumstances. It is a metal of spec. grav. 19.3, fusing at 2016° F., ductile, and so very malleable, that gold leaf may be made of only one two-hundred thousandth of an inch in thickness; but what pre-eminently fits it for use in the mouth is its great durability, and the fact that it is unaffected by air or water, single acids, alkalies, or sulphuretted hydrogen. For stopping teeth it should be pure, but for general dental purposes, it should be alloyed, not with copper, but with silver. Platinum—a white metal—closely resembles gold in most respects; it is less easily fused, has a spec. grav. of 21.5, is nearly as malleable, and is equally unaffected by such chemical agents as may find their way into the mouth. Palladium closely resembles platinum, and has been much used, but its price has greatly risen of late. Silver has a spec. grav. of 10.5, fuses at 1873°, is extremely ductile and malleable, and is unaffected by air or water. It is rather more easily attacked by other substances than gold is, but its chief disadvantage in the mouth is, that its beautiful white color is gradually changed to perfect blackness by the sulphur compounds in the food. Tin has the same advantages as gold or platinum, but in an inferior degree. Cadmium, another white metal, harder than tin, of spec. grav. 8.7, fusing at 442° and very malleable, has been used with some success. When aluminium, the white metal, of only 2.6 spec. grav., derived from clay, was lately produced in quantity, it was expected that it would prove valuable in the stopping of teeth; but though malleable and unaffected by acids, it is easily attacked by alkalies, and therefore by the saliva. Fusible alloys of bismuth, lead, and tin, have been recommended for stopping; but they can only be fused and put into the tooth at a temperature just under that of boiling water, and they have all the disadvantages of lead. There is an objection in many cases to metallic stoppings, arising from the easy conduction of heat or cold to the exposed nerve; but this may be obviated by interposing between the metal and the nerve, asbestos, horn, or some other non-conductor.

Dr. Gladstone then proceeded to describe rapidly the manufacture of artificial teeth. They are sometimes cut from the tusks of the hippopotamus, or the walrus; but in such a case, it must be remembered that the ivory is dead, and hence decomposition and blackening of the animal portion of the artificial tooth gradually ensues. Mineral teeth are made in very nearly the same way as porcelain, but they are not so brittle as that article. The materials employed in their manufacture are felspar, kaolin (decomposed felspar), some kinds of clay, silica (flint or quartz), and sometimes glass and borax; and for colouring—compounds of platinum, gold, manganese, cobalt, titanium, uranium, zinc, and silver. The processes of moulding the teeth, baking them so as to form a kind of biscuit ware, putting on the enamel, firing them in a muffle furnace, and annealing, were briefly recapitulated; the formation of mineral or ivory blocks, and of gold plates, was glanced at; as well as the materials used in modelling, viz., wax, plaster of Paris, metallic alloys (zinc, bismuth, and tin), and lead.

The lecturer explained that he had not time to enter upon mechanical details, nor yet upon many principles of natural philosophy that were of high interest to the dentist; nor had he been able to speak of chloroform, morphia, quinine, or other articles of materia medica, in this rapid introduction to chemistry in its applications to dental science and art.—*Quarterly Journal of Dental Science.*

